# Supplementary material for: Huygens metasurface supporting quasi-bound states in the continuum for terahertz gas sensing
Source: Sci Rep. 2025 Apr 25;15:14504. doi: 10.1038/s41598-025-99068-8 (PMC12032221; doi:10.1038/s41598-025-99068-8)
Supplement: Supplementary file 1 — Supplementary Information. [file 41598_2025_99068_MOESM1_ESM.pdf]

Huygens metasurface supporting quasi-bound states in the continuum for terahertz gas sensing

Jose Antonio Álvarez-Sanchis, Borja Vidal, Ana Díaz-Rubio

Supplementary information

1 Multipolar decomposition

Figure S1 shows the multipolar decomposition of the response of one of the resonators of the metasurface for both the uncoupled and coupled cases. It can be seen that there is no contribution from quadrupole modes and that the ED mode is the one with the higher quality factor. These values were obtained from applying the equations in [1] to the scattered fields from the COMSOL model used to simulate the resonances.

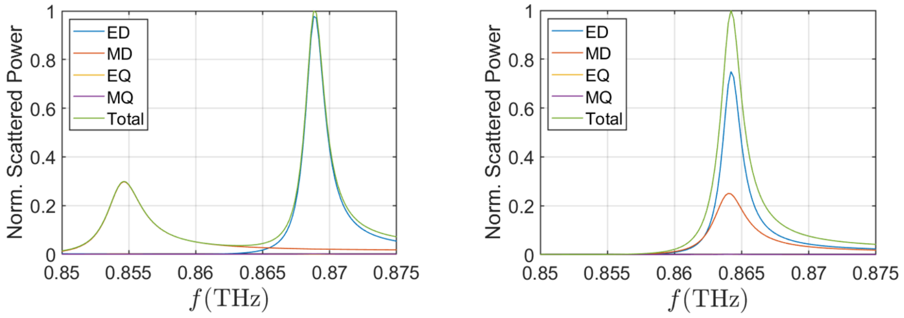

(a) **(b)**

S 1: Normalized scattered power by dipolar and quadrupolar modes as a function of frequency for (a) the uncoupled ($L = 226.65\mu$m, $w = 10.88\mu$m, $d_1 = 118.4\mu$m, $h = 60.5\mu m$ and $\Delta = 13.6\mu$m) and (b) the coupled ($L = 229.6\mu m$, $w = 10.88\mu$m, $d_1 = 119.9\mu$m, $h = 58.8\mu$m and $\Delta = 13.6\mu$m) cases.

2 Parameters for Figure 4(c)

Δ	L	d_1	h
2.09 μm	232.35 μm	127.26 μm	58.21 μm
3.02 μm	232.23 μm	126.73 μm	58.21 μm
4.18 μm	232.13 μm	126.10 μm	58.24 μm
6.26 μm	231.86 μm	124.90 μm	58.33 μm
8.56 μm	231.27 μm	123.43 μm	58.41 μm
10.83 μm	230.58 μm	121.91 μm	58.54 μm
13.60 μm	229.60 μm	119.90 μm	58.80 μm

References

[1] Alaee, R., Rockstuhl, C., and Fernandez-Corbaton, I. Exact Multipolar Decompositions with Applications in Nanophotonics, *Advanced Optical Materials*, vol. 7, no. 1, p.1800783, 2019, https://doi.org/10.1002/adom.201800783.
